# Shock Wave Response of Iron-based *In Situ* Metallic Glass Matrix Composites

**DOI:** 10.1038/srep22568

**Published:** 2016-03-02

**Authors:** Gauri R. Khanolkar, Michael B. Rauls, James P. Kelly, Olivia A. Graeve, Andrea M. Hodge, Veronica Eliasson

**Affiliations:** 1 Department of Aerospace and Mechanical Engineering, University of Southern California, Los Angeles, CA 90089, USA; 2 Graduate Aerospace Laboratories, California Institute of Technology, Pasadena, CA 91125, USA; 3 Department of Mechanical and Aerospace Engineering, University of California, San Diego, La Jolla, CA 92093-0411, USA; 4 Kazuo Inamori School of Engineering, Alfred University, Alfred, NY 14802, USA; 5 Mork Family Department of Chemical Engineering and Materials Science, University of Southern California, Los Angeles, CA 90089, USA

## Abstract

The response of amorphous steels to shock wave compression has been explored for the first time. Further, the effect of partial devitrification on the shock response of bulk metallic glasses is examined by conducting experiments on two iron-based *in situ* metallic glass matrix composites, containing varying amounts of crystalline precipitates, both with initial composition Fe_49.7_Cr_17.7_Mn_1.9_Mo_7.4_W_1.6_B_15.2_C_3.8_Si_2.4_. The samples, designated SAM2X5-600 and SAM2X5-630, are X-ray amorphous and partially crystalline, respectively, due to differences in sintering parameters during sample preparation. Shock response is determined by making velocity measurements using interferometry techniques at the rear free surface of the samples, which have been subjected to impact from a high-velocity projectile launched from a powder gun. Experiments have yielded results indicating a Hugoniot Elastic Limit (HEL) to be 8.58 ± 0.53 GPa for SAM2X5-600 and 11.76 ± 1.26 GPa for SAM2X5-630. The latter HEL result is higher than elastic limits for any BMG reported in the literature thus far. SAM2X5-600 catastrophically loses post-yield strength whereas SAM2X5-630, while showing some strain-softening, retains strength beyond the HEL. The presence of crystallinity within the amorphous matrix is thus seen to significantly aid in strengthening the material as well as preserving material strength beyond yielding.

Iron-based bulk metallic glasses (BMG), or amorphous steels, are known to have high hardness, high fracture strengths of 4 GPa or more, good corrosion resistance and magnetic properties, as well as the added advantage of lower material costs over other metallic glasses^1^,^2^,^3^,^4^,^5^,^6^. Even though Fe-BMGs have several attractive attributes, most of them have low toughness and are consequently extremely brittle and undergo catastrophic failure^1^, which severely limits their applications. The low toughness of Fe-BMGs has been associated with their high shear modulus and glass transition temperature, which result in high activation barriers for shear flow thereby limiting plastic deformation^7^,^8^. Subsequently, the development of different compositions by employing varying chemistries and alloying strategies that vary elastic constants, has lead to improved ductility and toughness^9^,^10^. In addition, iron based metallic glass matrix composites (MGMC) containing precipitated crystalline phases, through varying degrees of annealing or micro-addition of nanocrystallization inducing elements, have been studied and proven to enhance ductility^11^,^12^,^13^. One such study, for example, resulted in significantly improved plasticity of over 30 percent plastic strain due to the *in situ* α-Fe dendritic phase as opposed to only 3 percent plastic strain in monolithic samples^11^. The presence of either precipitated α-iron phase or iron-metalloid compounds, or a combination of both in the amorphous matrix is seen to result in increased plasticity or even higher hardness^14^,^15^.

In order to ensure the viability of amorphous steels for use in structural applications, it is necessary to characterize their mechanical response over a wide range of strain rates. Thus far, there have been several studies on the quasi-static mechanical response of amorphous steels and their composites^1^,^6^,^9^,^10^,^11^,^16^, however, to the best of our knowledge, their high strain-rate shock wave response has not been explored yet. High strain-rate mechanical response is relevant for applications such as kinetic energy penetrators, ballistic armor, and satellite shields for protection from meteorite impact. The shock response of bulk metallic glasses reported so far in the literature involves work on mainly Zr-based alloy systems, which have been shown to have a two-wave elastic-plastic response, high Hugoniot Elastic Limit (HEL) typically around 7 GPa and post-yield strain-softening under shock wave compression^17^,^18^,^19^,^20^,^21^,^22^,^23^,^24^,^25^. The only reported work thus far on the shock response of a MGMC involved a comparison of a monolithic Zr-BMG with its β -dendritic phase *in situ* composite containing 25 percent volume fraction crystallinity and revealed no significant difference between the response of the two under shock compression^26^.

In this work, we explore the shock wave response of two iron-based *in situ* metallic glass matrix composites referred to as SAM2X5-600 and SAM2X5-630 with the initial composition Fe_49.7_Cr_17.7_Mn_1.9_Mo_7.4_W_1.6_B_15.2_C_3.8_Si_2.4_, each containing a different amount of *in situ* crystalline precipitate. In addition to exploring the high strain rate response of amorphous steels, the aim of this work is to determine the effect of devitrification on the shock response, if any. Both sample types are prepared using the method of spark plasma sintering, starting from amorphous powders, and each has been sintered at different temperatures in the super-cooled liquid regime (temperature between the glass transition and crystallization temperature of a metallic glass), resulting in slight variations in the microstructure with respect to devitrification. The SAM2X5-630 samples were produced from sintering powders at 903 K (630 °C), which resulted in the precipitation of sub-micron sized (Fe,Cr,Mn,Mo,W)_23_(B,C,Si)_6_ crystalline regions scattered throughout the amorphous matrix. SAM2X5-600 was produced from sintering powders at a temperature below the onset temperature of crystallization detectable by X-rays and, as such, the samples are X-ray amorphous. The difference in extent of devitrification in the two sample types allows a systematic study of the effect of partial devitrification on the mechanical response of these composites at high strain rates. Plate impact experiments, involving the launching of a flyer plate onto a stationary target, are used to generate normal shock waves in the sample, with velocity interferometry being the primary diagnostic.

## Results

A summary of measured ambient mechanical properties of the samples SAM2X5-600 and SAM2X5-630 is presented in Table 1. Conditions ahead of and behind a shock wave traveling in a medium are related through conservation equations known as the Rankine-Hugoniot jump conditions. The equations for conservation of mass, momentum and energy for a shock propagating into a material in the ambient state are given as follows^27^:


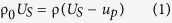



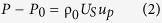



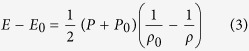


**Figure 1:**
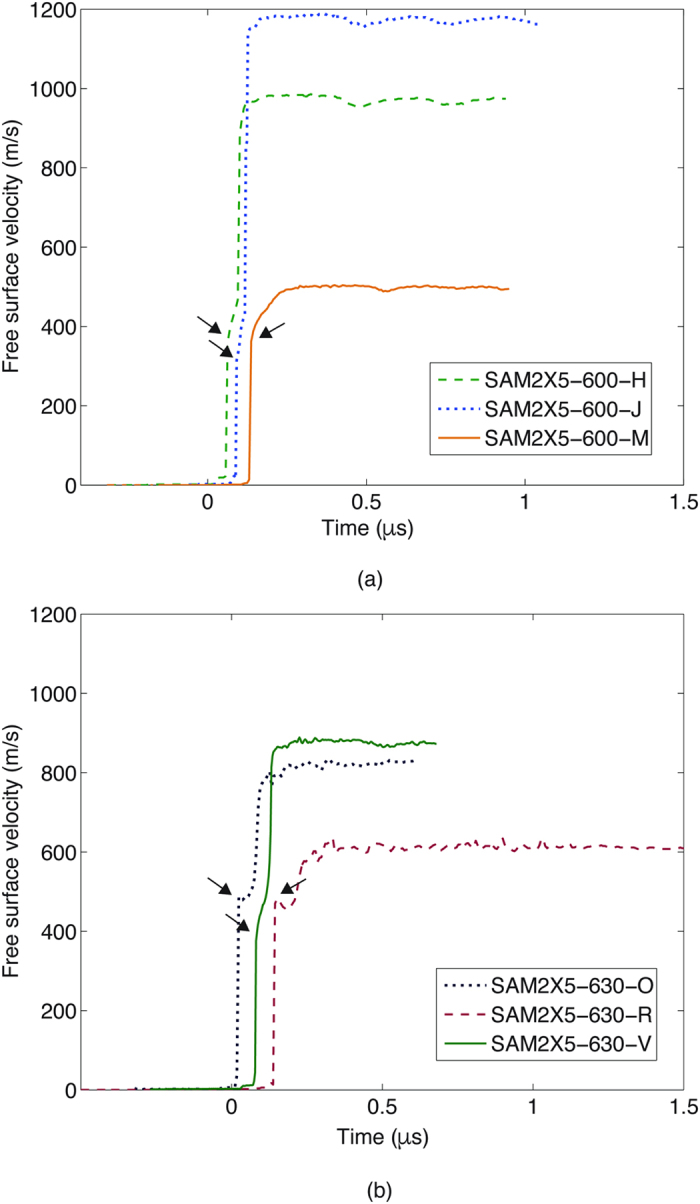
Wave profile data showing measured free surface velocity at sample rear surface for (**a**) SAM2X5-600 and (**b**) SAM2X5-630. Arrows on the plot indicate the HEL point.

Here *P, E, U*_*S*_, *u*_*p*_ and ρ are the pressure, internal energy, shock velocity, particle velocity and density respectively. The 0 subscript indicates quantities in the ambient state. The Rankine-Hugoniot conservation equations assume the existence of a discontinuous, steady wave^27^. Since there are five thermodynamic variables and three conservation equations, the measurement of any two allows the calculation of the remaining variables. The particle velocity and shock velocity have been measured in these experiments using velocity interferometry and time of arrival measurements, respectively. Particle velocity histories measured at the rear free surface of both sample types are shown in Fig. 1. Each of the wave profiles has a two-wave structure, consisting of an elastic precursor wave followed by a slower moving plastic shock wave, indicating elastic-plastic deformation before reaching the peak Hugoniot state. Arrows pointing at the knee in the wave profiles indicate the HEL point. This data has been analyzed to determine shock velocities, HEL state and the peak state.

### Calculation of HEL and peak states

Elastic shock velocities are calculated using transit time of the shock through the sample from experimentally measured arrival times at the front and back of the sample, and the known sample thickness. The in-material particle velocity is obtained from the measured free surface velocity using the free surface approximation^28^, as follows:


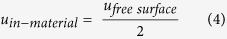


The particle velocity at the HEL is identified at the intersection of the two lines that are fit to the two legs of the knee corresponding to the HEL point in the measured wave profile. Using the elastic shock velocity *U*_*sh,el*_ and particle velocity at the HEL *u_HEL_*, the HEL stress may be computed as follows:


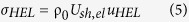


Here, ρ_0_ is the ambient density of the sample. The density of the sample after passage of the elastic precursor, ρ*_HEL_*, is determined from the first Rankine-Hugoniot jump condition as follows:


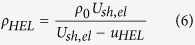


The density in the peak shocked state, ρ*_peak_*, may be determined in a similar manner using the plastic shock speed *U*_*sh,pl*_ and peak particle velocity *u_peak_*, as follows:


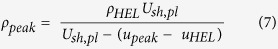


The Eulerian plastic shock speed is calculated after determining the transit time of the plastic wave after precursor arrival to the mid-point of the plastic wave rise δ*t* as follows:


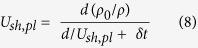


Here, *d* is the original thickness of the sample. Finally, the stress in the peak shocked state of the sample is calculated using the second Rankine-Hugoniot jump condition as follows:


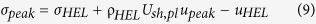


A summary of calculations performed using measurements for both SAM2X5-600 and SAM2X5-630 is presented in Tables 2 and 3. SAM2X5-630 samples used in experiments are designated SAM2X5-630-O, SAM2X5-630-R, SAM2X5-630-V while the SAM2X5-600 samples are represented by SAM2X5-600-H, SAM2X5-600-J, SAM2X5-600-M. SAM2X5-600 samples were subjected to stresses in the range 11–25 GPa corresponding to impact from copper flyer plates at velocities between 500 and 1300 m/s. SAM2X5-630 samples were also impacted using copper flyers at loading stresses of 14–19 GPa corresponding to impact velocities between 700 and 1000 m/s. Strain rates for all the experiments ranged from 6 × 10^5^–2.5 × 10^6^ per second, and were evaluated from the fastest rising portion of the shock wave. SAM2X5-600 and SAM2X5-630 are seen to have HELs of 8–9 GPa and 10–12.5 GPa respectively, which corresponds to elastic strains as large as about 3% and 4% respectively. These are higher than elastic limits of about 7 GPa previously reported for metallic glasses of other compositions under shock loading^17^,^18^,^19^,^22^,^23^,^24^,^25^. The latter especially is considerably higher, and is nearly 1.5 times that of previous results for the HEL of a metallic glass. The elastic limit measured for SAM2X5-630 is comparable to that of other high-strength hard, brittle ceramics such as SiC and TiB_2_^29^.

### Calculation of hydrostat

A high HEL is indicative of the energy-absorbing ability and therefore the shock-resistance of a material and is seen to be a benchmark for ballistic performance. However, the elastic limit alone may not be a good indicator of material strength and performance. The offset between static isothermal data and shock data is, therefore, commonly used to assess the shear strength achieved in the shock-compressed state^30^. For this purpose, the static data may be obtained from hydrostatic experiments, or it may be computed from data from shock experiments if lateral stress measurements are made in addition to the usual longitudinal stress measurements. In the absence of experimental hydrostatic data however, the hydrostat may be analytically calculated while making some assumptions. In this work, the shear modulus is assumed to remain constant in order to calculate the mean stress. Elastic shock velocity from each experiment is plotted against particle velocity corresponding to the elastic limit. A linear fit through this data results in an equation of the form:


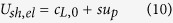


Here, *c*_*L*,0_ is the ambient longitudinal sound speed measured using the ultrasonic pulse echo technique and the parameter *s* is the slope of the linear fit. This equation is converted to the stress-density compression (σ − μ) plane using the Rankine- Hugoniot jump conditions^27^ and is expressed as^17^:


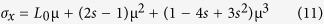


Here, *L*_0_ is the ambient longitudinal modulus. Equation (11) represents the longitudinal elastic stress as a function of density compression. In order to make inferences about the strength of the material, a comparison of the Hugoniot and the hydrostat is necessary. The hydrostat represents the average of stresses in the three principal directions i.e. the longitudinal stress (measured here) and two lateral stresses. Following the framework for the calculation of the hydrostat as laid out by Fowles^31^, the shear modulus *G* is assumed to remain constant, since lateral stresses were not measured. The mean stress σ_*m*_ is then calculated from the longitudinal stress as follows:


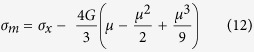


**Figure 2:**
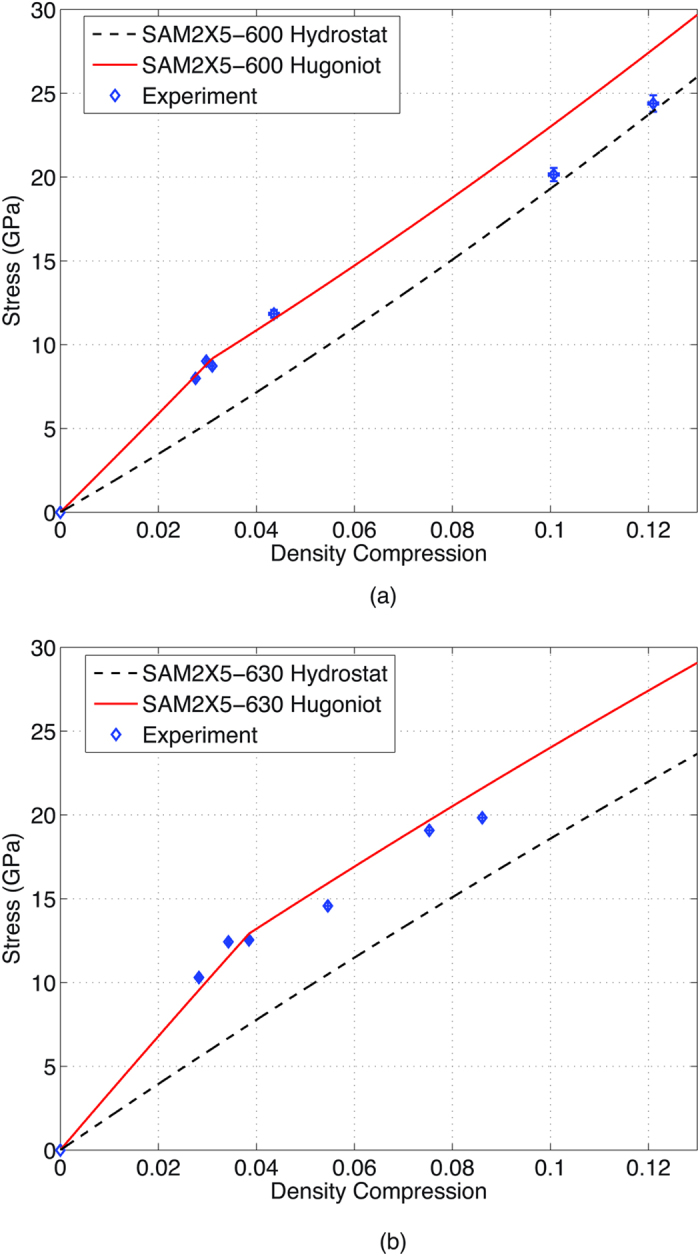
A comparison of calculated Hugoniot (solid line), hydrostat (dashed line) and experimental data (solid diamond marker) for (**a**) SAM2X5-600 and (**b**) SAM2X5-630. Error bars represent calculated uncertainty in stress and density compression, propagated from uncertainty in density, particle velocity and shock velocity measurements.

The mean stress calculated from Equation (12) thus represents the analytical hydrostat for the material, whereas Equation (11) represents the elastic Hugoniot. The Hugoniot beyond the elastic limit is constructed assuming elastic-perfectly plastic material response by adding the constant offset value of 2*Y*/3, *Y* being the quasi-static yield strength, to the calculated hydrostat. Quasi-static studies of amorphous alloys of various compositions have previously indicated elastic-perfectly plastic behavior^32^,^33^. In addition, shock wave calculations using the elastic perfectly-plastic assumption have matched experimental wave profile data in previous studies^17^,^18^. Therefore, this type of material response is an appropriate assumption for material behavior beyond the elastic limit for the amorphous alloys of this work. The experimentally obtained stresses and density compressions are plotted alongside the calculated Hugoniots and hydrostat to evaluate the strength of the material, as seen in Fig. 2. It can be seen that while both sample types strain-soften beyond the elastic limit, SAM2X5-600 seems to dramatically lose shear strength at high loading stresses. On the other hand, SAM2X5-630 seems to retain some post-yield strength. Calculations for estimation of temperature rise within the shock front using the measured equation of state resulted in a final temperature of 438 K in SAM2X5-600 at the maximum loading stress of 25.13 GPa and 423 K in SAM2X5-630 at 19.83 GPa. Both of these temperatures are significantly lower than the reported glass transition temperature of 850 K for the chemical composition of SAM2X5-600 and SAM2X5-630^34^, as well as the temperatures associated with long-range ordering and devitrification^35^. The shock response of both composites is therefore not influenced by any potential phase transitions induced by the temperature rise within the shock wave, since these likely do not occur under the range of impact stresses examined here. An assumption of the existence of steady waves in samples of both composites is implicit in the analysis presented above. However, further experiments to examine wave traces at a given impact stress for samples of widely varying thicknesses are needed to verify this assumption.

## Discussion

While the mechanistic phenomena behind the elastic limit and yielding of crystalline metals under shock loading is fairly well understood in terms of dislocation-mediated slip, the physical interpretation of the elastic limit in a brittle amorphous solid, such as the iron-based BMGs studied in this work, can be understood as the onset of relieving of shear strains from fracture by the joining and interaction of damaged zones and subsequent flow of the material^36^. In general, various mechanisms by which slip, and thereby yielding, may occur to accommodate shear strains have been proposed for brittle solids; these include plastic-brittle deformation comprising of slip zones with cracks and micro-cracks, brittle destruction deformation consisting of cracks and cleavages, and deformation from partial-melting zones^29^.

A system of classification for the yielding of shocked solids based on the offset of the Hugoniot from the hydrostat or the isotropic loading state beyond the elastic limit has been described previously^28^,^29^. Following this system, materials may be classified into three main categories based on the comparison of their response to shock and isotropic loading when considered in the stress-density compression space: toughening solids whose shear strength increases with applied stress, perfect elasto-plastic solids which maintain a constant offset between their Hugoniot and hydrostat, and perfect elasto-isotropic solids which catastrophically lose shear strength above the HEL with the stress collapsing onto the isotropic state. Material behavior often lies between these two idealized extreme scenarios, and this is seen in the material response of SAM2X5-600 and SAM2X5-630 as is apparent in Fig. 2. For both materials, the data points clustered around the inflection in the Hugoniot correspond to the HEL. For the SAM2X5-600 samples, the data point corresponding to the peak state at the lowest impact stress of 11.84 GPa seems to be in accordance with the elasto-plastic calculation. However at the higher impact stresses, both data points lie very close to the hydrostat, thus suggesting that there exists a certain threshold between 12 and 20 GPa beyond which SAM2X5-600 catastrophically loses all strength with the Hugoniot consequently nearly collapsing onto the isotropic stress state. Such catastrophic loss of shear strength has also been observed in high-HEL, brittle and hard materials such as boron carbide and silicon nitride^37^. On the other hand, peak state measurements for SAM2X5-630 compared to its calculated Hugoniot suggest that although the material strain-softens it seems to retain its strength even at impact stresses nearly one and a half times its HEL. This observed loss of post-yield strength in SAM2X5-630 and SAM2X5-600 is likely a result of yielding phenomena arising out of slip from propagation of cracks, cleavage and partial melting. Under intense loading scenarios such as the ones shock compression experiments present, the free volume in the amorphous matrix is quickly depleted and large stress concentrations are accumulated at those sites. Micro-voids coalesce into shear bands, which then provide pathways for the propagation of micro-cracks arising out of areas of intense shear localization. Evidence for partial strength loss beyond the elastic limit has also been seen in previous shock studies of various Zr-based metallic glasses^17^,^18^,^19^,^20^,^21^ where maximum shock stresses ranged from just above the HEL to an order of magnitude higher than the HEL.

**Figure 3:**
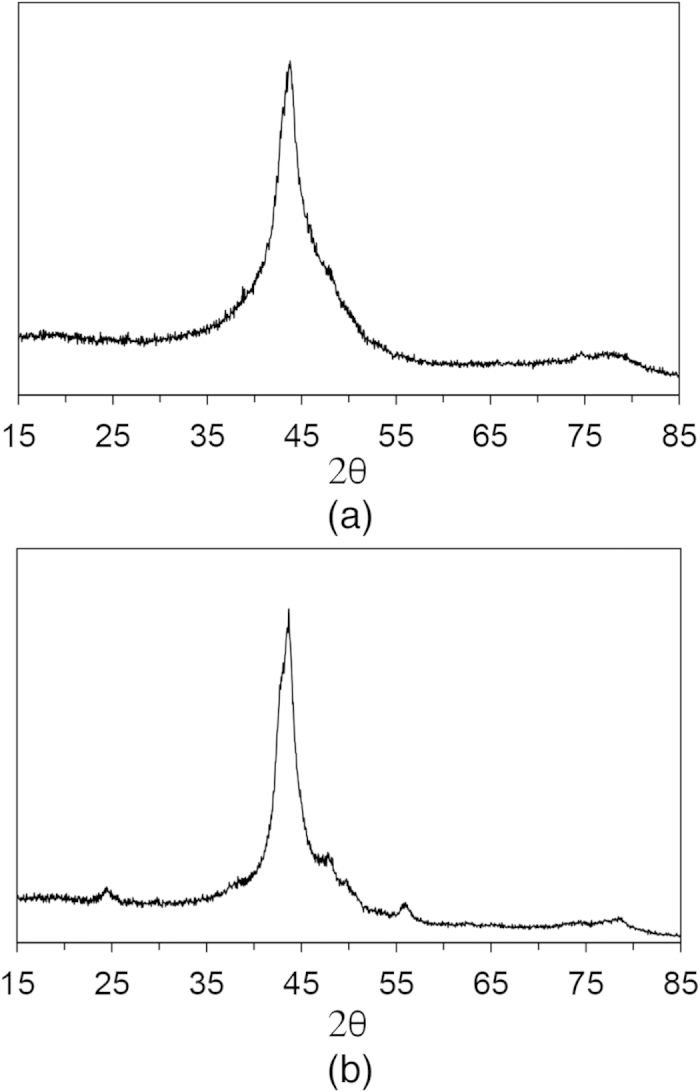
X-ray diffraction patterns of (**a**) SAM2X5-600 and (**b**) SAM2X5-630. The sharper peak of sample SAM2X5-630 is an indirect measure of the higher extent of crystallinity in this sample.

Since the maximum shock stress that SAM2X5-630 was subjected to is less than the maximum impact stress for SAM2X5-600, a further loss of strength for SAM2X5-630 than is suggested by the current data cannot be completely ruled out. Even then, such dramatically different responses at high Hugoniot pressures within a comparable range of impact stresses for samples that are nearly identical in their make-up, except for a very small amount of crystallinity, cannot be ignored. X-ray diffraction patterns of the samples can be seen in Fig. 3. As can be seen, sample SAM2X5-600 (Fig. 3(a)) is X-ray amorphous, whereas sample SAM2X5-630 (Fig. 3(b)) contains regions of nanocrystallinity. In sintering samples of SAM2X5-630, the amorphous powders were sintered at 903 K, which is above the temperature at which structural transformation corresponding to the crystallization of (Fe,Cr,Mn,Mo,W)_23_(B,C,Si)_6_ occurs^35^. On the other hand, samples of SAM2X5-600 were made by sintering powders at 873 K, just below the onset of this crystallization, but above the temperature at which structural relaxation and some long-range ordering occurs. Metal carbides and borides are known to have high hardness values, and the presence of the nanocrystallites dispersed in the amorphous matrix within SAM2X5-630 are likely responsible for strengthening this material - in both increasing its elastic limit compared with SAM2X5-600, as well as retention of post-yield strength - by acting as barriers to the propagation of failure fronts in the form of shear bands and cracks. In a previous work, annealing of an Fe-Co-B-Si-Nb metallic glass resulted in enhanced hardness and Young’s modulus, with mechanical hardening being attributed to the precipitation of the metastable Fe_23_B_6_ phase^15^, which is believed to be present in SAM2X5-630 as well^35^. In another work, the precipitation of the Fe_23_B_6_ phase in a Fe-Dy-B-Nb metallic glass was also seen to enhance fracture strength and Vickers hardness, in addition to improving the thermal stability and glass forming ability of the composition^38^. While a slightly higher Young’s modulus (as a result of larger sound speeds) is observed in SAM2X5-630 as compared to SAM2X5-600, no significant difference in Vickers hardness is observed between the two sample types, as seen in Table 1. Therefore, while a small proportion of crystalline precipitate is not large enough to cause any observable distinction in the ambient and quasi-static mechanical response of SAM2X5-630 and SAM2X5-600, it proves to be significant in influencing the response of the shocked samples at very high strain rates (of the order of 10^6^ per second).

In conclusion, amorphous steels, the high-strain rate mechanical response of which was hitherto unexplored, demonstrate high strength under shock wave compression, the magnitude of the elastic limit of one amorphous steel composite being 1.5 times those reported previously for amorphous metals. Further, a minor addition of nanocrystallinity to the amorphous matrix of the iron-based BMG studied in this work results in a significant improvement in yield strength and post-yield shear strength retention, as seen in the response of the partially crystalline SAM2X5-630 when compared with that of the X-ray amorphous SAM2X5-600. This result is in contrast with a previous work on Zr-BMG Vitreloy 1 and its *in situ* dendritic phase composite, where no difference was seen in the shock response of the monolithic BMG and its composite^26^. While the shock response of the amorphous steels of this work is qualitatively similar to that of previously studied Zr-based compositions, namely large amplitude elastic waves and loss of post-yield strength, a significant enhancement in response based on the extent of devitrification is observed in both of these attributes. This work therefore reveals *in situ* reinforced metallic glass composites as promising candidates for use in high-strength applications. Furthermore, they demonstrate that controlled devitrification is a potentially viable adjustable parameter for synthesis of amorphous metallic materials with properties that can be tailored as desired.

## Methods

### Sample Preparation and Characterization

Samples were prepared using spark plasma sintering^39^,^40^,^41^,^42^, described previously^34^,^35^. Briefly, the samples were prepared by consolidating SAM2X5 metallic glass powders inside a graphite die that produces cylindrical samples with diameters of 19 mm. The model for the spark plasma sintering unit is the HP D25 from FCT Systeme. Sample SAM2X5-600 was heated to a temperature of 873 K (600 °C) with no hold time at temperature. Sample SAM2X5-630 was heated to a temperature of 903 K (630 °C) with no hold time at temperature. The heating rate from room temperature to the sintering temperature was 500 K/min for both types of samples.

All BMG samples were characterized for density using the Archimedean method using an Ohaus Solids Density Determination Kit, Vickers hardness on a Leco LM100 system using a 300 gram-force held for 10 seconds, X-ray diffraction on a Bruker D2 Phaser instrument using Cu Kα radiation, and longitudinal and shear sound speed measurements using ultrasonic transducers and the pulse-echo technique using an Olympus 38DI Plus Ultrasonic Thickness Gauge.

### Shock Compression Experiments

Shock waves were generated in the sample by the plate impact technique in which a flyer plate is launched at high velocities using a propellant gun onto a target sample. The iron-based BMG samples were nominally 19 mm in diameter and 1.5–1.8 mm in thickness. The flyer plates were made of OFHC copper and were nominally 34 mm in diameter and 1 mm in thickness. All target and flyer plates were lapped flat and parallel to within 2 microns to ensure a normal shock wave. Flyer plates were mounted onto Nylatron sabots 45 mm long with a cavity at the front end to accommodate the copper flyer plate and were launched using a naval powder gun. Flyer plates were epoxied into the sabot using Hysol Loctite E-20 HP epoxy and were cured under a flat steel weight overnight. Lapped BMG samples were epoxied into a polycarbonate target holder, with a ridge for sample placement at the center and hole placements for shorting pins (Dynasen CA-1038) and interferometry probes (AC Photonics 1CL15P006LCC01). Particle velocity as well as projectile impact velocity was measured using a custom- built Photonic Doppler Velocimetry (PDV) system. The PDV system is a heterodyne PDV that allows for four simultaneous velocity measurements by splitting the laser light (NKT Photonic 2 W drive laser with a 20 mW reference laser) four ways into four PDV probes. In the experiments of this work, only two probes were used, one focused on the flyer plate for projectile velocity measurement, and the other focused at nominally the center of the rear surface (non-impact side) of the metallic glass sample for particle velocity measurement. Data acquisition is carried out through an Agilent MSO 9104A oscilloscope with a sampling rate of 20 GS/s. As part of target preparation before each experiment, PDV probes are set into the target holder at a height that results in the least possible return loss, monitored by a hand-held optical return loss meter (JDSU SmartClass ORL-55). Once targets were completely assembled, the probes were affixed to the PDV system while making sure that the fiber ends were clean by examining through a fiber scope. Attenuators on the PDV system were adjusted to tune the laser return from the target. Finally, recorded fringe data were reduced to particle velocity wave profiles using the PlotData software from Sandia National Labs.

## Additional Information

**How to cite this article**: Khanolkar, G. R. *et al*. Shock Wave Response of Iron-based *In Situ* Metallic Glass Matrix Composites. *Sci. Rep.* **6**, 22568; doi: 10.1038/srep22568 (2016).

## Figures and Tables

**Table 1:** Material characterization results.

Sample	Density (g/cc)	Longitudinal Sound Speed (km/s)	Shear Sound Speed (km/s)	Vickers Hardness (GPa)
SAM2X5-600	7.75 ± 0.04	6.12 ± 0.04	3.42 ± 0.06	16.16 ± 0.54
SAM2X5-630	7.87 ± 0.02	6.61 ± 0.04	3.68 ± 0.02	16.34 ± 0.50

**Table 2:** Summary of calculated SAM2X5-600 results.

	SAM2X5-600-H	SAM2X5-600-J	SAM2X5-600-M
Impact Velocity (km/s)	1.104 ± 0.002	1.300 ± 0.002	0.584 ± 0.001
Elastic Shock Velocity (km/s)	6.20 ± 0.02	6.20 ± 0.02	6.40 ± 0.02
Plastic Shock Velocity (km/s)	4.72 ± 0.10	5.09 ± 0.10	5.05 ± 0.10
HEL Particle Velocity (km/s)	0.182 ± 0.009	0.167 ± 0.009	0.181 ± 0.009
HEL (GPa)	8.73 ± 0.13	7.99 ± 0.12	9.02 ± 0.14
Elastic Density Compression	0.0301 ± 0.003	0.0276 ± 0.003	0.0292 ± 0.003
Peak Particle Velocity (km/s)	0.485 ± 0.009	0.590 ± 0.009	0.251 ± 0.009
Peak Density Compression	0.1007 ± 0.010	0.1209 ± 0.012	0.0436 ± 0.004
Peak Stress (GPa)	20.15 ± 0.71	25.13 ± 0.89	11.84 ± 0.41

**Table 3:** Summary of calculated SAM2X5-630 results.

	SAM2X5-630-O	SAM2X5-630-R	SAM2X5-630-V
Impact Velocity (km/s)	0.705 ± 0.001	0.918 ± 0.002	1.001 ± 0.002
Elastic Shock Velocity (km/s)	6.53 ± 0.02	6.59 ± 0.02	6.88 ± 0.02
Plastic Shock Velocity (kms)	4.15 ± 0.10	4.82 ± 0.10	4.69 ± 0.10
HEL Particle Velocity (km/s)	0.242 ± 0.009	0.242 ± 0.009	0.189 ± 0.009
HEL (GPa)	12.43 ± 0.19	12.54 ± 0.19	10.30 ± 0.15
Elastic Density Compression	0.0385 ± 0.004	0.0382 ± 0.004	0.0283 ± 0.003
Peak Particle Velocity (km/s)	0.306 ± 0.009	0.409 ± 0.009	0.439 ± 0.009
Peak Density Compression	0.0546 ± 0.005	0.0753 ± 0.008	0.0861 ± 0.009
Peak Stress (GPa)	14.57 ± 0.51	19.08 ± 0.67	19.83 ± 0.69
